# Open Reduction and Internal Fixation with Mini-plate and Screws for Management of Unstable Metacarpal Fracture among Hand Injuries in a Tertiary Care Center: A Descriptive Cross-sectional Study

**DOI:** 10.31729/jnma.6846

**Published:** 2021-07-31

**Authors:** Sagar Panthi, Rishiswor Shrestha, Jigyasu Pradhan, Bikash Neupane, Siddhartha Khanal, Angelica Karki, Deepa Sharma

**Affiliations:** 1 Department of Orthopaedics and Traumatology, Rapti Academy of Health Sciences, Ghorahi, Dang, Nepal; 2 Department of Orthodontics and Dentofacial Orthopaedics, Rapti Academy of Health Sciences, Ghorahi, Dang, Nepal; 3 Department of Community Medicine and Public Health, Rapti Academy of Health Sciences, Ghorahi, Dang, Nepal

**Keywords:** *fracture fixation*, *metacarpal bones*, *mini-plate*

## Abstract

**Introduction:**

Hand injuries metacarpal fractures are common and it accounts about 14 to 28%. Mini-plate fixation in unstable metacarpal fractures provides absolute stability and early mobilization of fingers to reduce complications. The purpose of this study is to find out the prevalence of open reduction and internal fixation with mini-plate and screws for management of unstable metacarpal fracture among hand injuries done in a tertiary care center.

**Methods:**

This was a descriptive cross-sectional study done from February 2019 and January 2021 in a tertiary care center with unstable isolated metacarpal fracture treated with mini-plate fixation and were followed up for six months duration. Ethical approval and informed written consent were taken from all patients. The outcome was assessed by the American Society for Surgery of the Hand Total Active Flexion Score. Convenient sampling method was used. Point estimate at 95% Confidence Interval was calculated along with frequency and proportion for binary data. Statistical Package for Social Sciences used for analysis.

**Results:**

Out of 250 patients who underwent hand surgeries, open reduction and internal fixation with mini-plate and screws for unstable metacarpal fracture were done in 32 (12.8%) (8.66-16.94 at 95% Confidence Interval). The mean time of fracture union was 6.78±1.008 weeks. Functional outcome according to American Society for Surgery of the Hand Total Active Flexion score was excellent in 25 (78.2%), good in 6 (18.8%), and poor in 1 (3%) patient.

**Conclusions:**

Fixation of metacarpal fracture by mini-plate and screws was required in fewer patients. Mini-plate fixation provides better stability and early mobilization for unstable metacarpal fractures to achieve a good functional outcome.

## INTRODUCTION

Among hand injuries metacarpal fractures are common and it accounts about 14 to 28% respectively.^1^ These types of injuries occur due to road traffic accidents, physical assault, and machinery injuries.^2^

Surgical management is one of the best options for unstable metacarpal fractures for good functional outcome.^3,4^ K-wire fixation either by closed or open technique is used for the management of metacarpal fractures. But these fixation devices have complications like finger stiffness, pin-tract infection and migration of K-wire. It maintains the length and alignment but rotations are not controlled by this method.^5-6^

Open reduction and internal fixation with mini-plate and screws which provides rigid fixation and allows early digital rehabilitation is preferred by various authors.^7,8^ This study aims to find out the prevalence of open reduction and internal fixation with mini-plate and screws for management of unstable metacarpal fracture among hand injuries done in a tertiary care center.

## METHODS

A descriptive cross-sectional study was carried out at Rapti Academy of Health Sciences, Ghorahi, Dang from period February 2019 to January 2021. Ethical approval was obtained from the IRB and informed written consent was taken from all patients. All 250 patients undergoing hand surgeries were included in the study. All patients with unstable metacarpal fractures who were treated with open reduction and internal fixation with mini-plate were followed up. Intra-articular fractures open fracture, multiple metacarpal fractures, age less than 18 and above 60 years were excluded from our study. A convenient sampling method was used. The sample size was calculated using a formula according to the study done by Nakashian M, et al.^9^

n = Z^2^ × p × q / e^2^

  = 1.96^2^ × 0.13 × 0.87 / 0.05^2^

  = 173

Where,

n = minimum required sample size
Z = 1.96 at 95% Confidence Interval (CI)
p = prevalence of open reduction and internal fixation^9^
q = 1-p
e = margin of error, 5%

The required sample size was 173. However, total sample size taken was 250 as we used convenient sampling technique.

The surgery for all patients was done under regional anesthesia. Pre-operatively one gram of intravenous Ceftriaxone was given thirty minutes before surgery. Tourniquet was applied and a dorsal approach was used to fix the fracture by 2.0 mm mini-plate and screws. Post-operatively patients were kept in a below-elbow volar slab. Passive mobilization was done on the third day and active mobilization was started on the second week after slab removal. The outcome was assessed by ASSH (American Society for Surgery of Hand and Total Active Flexion) score^10^ at six months follow-up. Statistical analysis was done by calculating mean and standard deviation via Statistical package for Social Sciences (SPSS) software version 21.0. Point estimate at 95% Confidence Interval and descriptive frequency was calculated.

## RESULTS

Out of 250 patients who underwent hand surgeries in our center, open reduction and internal fixation with miniplate and screws for unstable metacarpal fracture were done in 32 (12.8%) (8.66-16.94% at 95% Confidence Interval). Among 32 patients mean age of patients was 37.78±8.31 ranging from 20-60 years, with a higher frequency of male 20 (62.5 %) than female 12 (37.5 %). The right side was more involved than the left side. Fall was the most common mechanism of injury. The transverse pattern of fracture was more common (Table 1). The mean time of fracture union was 6.78±1.088 weeks.

**Table 1:** Demography of study participants (n = 32).

Characteristics	n (%)
**Sex**	
Male	20 (62.5)
Female	12 (37.5)
Side	
Right	21 (65.6)
Left	11 (34.4)
Mechanism of Injury	
Fall	15 (46.9)
Physical assault	10 (31.3)
Road Traffic Accident	7 (21.8)
Fracture Pattern	
Transverse	13 (40.6)
Oblique	6 (18.8)
Spiral	7 (21.8)
Comminuted	6 (18.8)
Age (Years)	37.78±8.31
Mean time of fracture union (months) 6.78±1.088	

Functional outcome at final follow-up by ASSH-TAF score was excellent in 25 (78.2%) of cases, good in 6 (18.8%) of cases, and poor in 1 (3%) of cases (Table 2). Two patients developed superficial wound infection which resolves with oral antibiotics. In one patient we couldn't achieve a good functional outcome and developed finger stiffness. We didn't encounter complications like implant failure, malunion, non-union, and complex regional pain syndrome.

**Table 2:** Functional outcome at final follow-up by Aerican Society for Surgery of Hand and Total Active Flexion (ASSH-TAF) score (n=32).

Characteristics	n (%)
Excellent	25 (78.2)
Good	6 (18.8)
Poor	1 (3)

## DISCUSSION

Metacarpal fractures can be managed conservatively in many cases if the fracture pattern is stable but unstable fractures warrant surgery.^11^ These fractures if not treated properly, may lead to loss of function of the hand as well as reduce the quality of life.^12,13^

Various fixation devices are used to manage a metacarpal fracture that includes K-wire fixation, tension band wiring, lag screw fixation. These fixation devices are not very stable. Fixation of unstable metacarpal fractures with mini-plate provides absolute stability which allows early mobilization of finger and prevents complications.^1,14^ In 1928, Lambotte described various surgical methods for the management of metacarpal fractures. It includes K-wire fixation, external fixators, a lag screw, and circlage wiring.^9^

Shehadi SI reported 100% functional outcome in metacarpal fractures managed by external fixators.^15^ Dreyfuss D, et al. did a non-randomized controlled trial study by using K-wire versus locking plate for management of metacarpal fractures in adults. Their result shows a good outcome with locking plate fixation as compared to K-wire fixation.^16^ Cha SM, et al. did a Quasi-experimental study comparing miniopen antegrade intramedullary nailing versus mini-plate fixation and the result was better in the mini-plate fixation group.^17^ Vasilakis V, et al. did a retrospective study comparing closed reduction and percutaneous K-wire fixation and mini-plate fixation and their results were not significant between two different modes of treatment.^18^ Pandey R, et al. did a randomized controlled trial study comparing closed reduction and percutaneous K-wire fixation with mini-plate fixation. On assessment at one year closed reduction percutaneous K-wire fixation had better results than mini-plate fixation.^19^ Chand P, et al. did a prospective study and concluded that 87.5% had excellent results with low profile mini-plate fixation.^20^

There were 62.5% male and 37.5% female. The most common mode of injury was fall which accounted for 46.9% of the total injuries. There was a significant relationship between gender and mode of injury. Physical assault was seen more in males this may be due to their more violent nature. Fifth metacarpal fractures were common. In the study done by Chand P, et al. third and fourth metacarpal fractures were common.^20^ Meantime of fracture union was 6.78±1.008 weeks which was comparable to study done by Basar H, et al.^21^

We could achieve excellent results in 78.2%, good in 18.8%, and fair in 3%. We achieved union in all cases. In one case we had poor results due to several factors such as a high degree of comminution of the fracture, post-operative superficial wound infection, and lack of compliance by the patient.

Two patients developed superficial wound infections which resolved with oral antibiotics. We didn't encounter complications like implant failure, malunion, non-union, and complex regional pain syndrome. Our sample size was small and 72.5% of patients belonged to the 21- 40 years age group which was comparable to the study done by Chand P, et al.^20^

## CONCLUSIONS

Fixation of metacarpal fracture by mini-plate and screws was required in fewer patients. It can be a good option for treating such fractures. More studies are needed to clarify the difference in fixation with other implants in unstable metacarpal fracture. The ultimate goal is to achieve excellent to good functional outcomes and early union of the fracture.
